# Abstracts

**DOI:** 10.1155/S1064744901000291

**Published:** 2001


### Symptoms associated with chlamydial infection among minority women

*T. S. Wen MD, J. M. Piper MD, J. E. Korte PhD, R. N. Shain PhD, E. R. Newton MD, S. Perdue DrPH, J. D. Champion PhD and A. E. C. Holden MA*

*Department of Obstetrics and Gynecology, The University of Texas Health Science Center at San Antonio, San Antonio, Texas*

**Objectives:** To assess the patterns of reported symptoms and signs in women infected with *Chlamydia trachomatis* alone (CT) *vs. Chlamydia* with bacterial vaginosis (BV), *Candida* (CA), gonorrhea (GC) or *Trichomonas* (TR) infection *vs.* other infections (BV, CA, GC, TR) without *Chlamydia*.

**Study design:** 602 African- and Mexican-American women with positive tests for GC (Gen-Probe), CT (Gen-Probe) or TR (culture) were enrolled in a randomized trial of a behavioral-cognitive intervention to reduce STD recurrence. Each woman underwent extensive questioning regarding genito-urinary symptomatology and had a standardized physical exam, including testing for common genital pathogens. Genito-urinary symptoms (patient report) and signs (clinician exam) were compared between women with CT only, CT+ (CT plus BV, CA, GC or TR) and CT- (BV, CA, GC or TR without CT). Categories of disease were initially analyzed separately and were collapsed based on the homogeneity of results. We present here only baseline data, at which time all the women were infected.

**Results:** Genito-urinary symptoms were commonly reported by women with CT only ($n = 120$), CT+ ($n = 301$) and CT- ($n = 181$) (Table 1). Pain symptoms were common: abdominal pain (38–42%), pain with sex (11–19%) and pain with urination (17–22%).

Genito-urinary symptoms were severe enough to cause the woman to take an action (stop having sex, douche, use medicine, or go to doctor) in 44% of CT only, 47% of CT+ and 47% of CT- cases.

**Conclusions:** A significant portion of women with genital infections report genito-urinary symptoms when specifically questioned. In contrast to other cross-sectional studies describing chlamydia as an asymptomatic infection, in our population women with chlamydial infection alone reported genito-urinary symptoms comparable to those in women with other vaginal infections.

**Table 1** Genito-urinary symptoms reported

| | *Discharge* | | | | | |
|---|---|---|---|---|---|---|
| | *Hx Mod/Heavy* | *Hx Yellow/Grn* | *Exam-Vag.* | *Exam-Cx* | *Hx Vaginal Itch* | *Hx Vaginal Odor* |
| CT only | 59.7% | 55.1% | 83% | 45.8% | 34.2% | 31.6% |
| CT+ | 64% | 54.6% | 87.9% | 41.2% | 36.4% | 42.2% |
| CT– | 64.7% | 47.8% | 86.2% | 38.1% | 40.6% | 42.6% |

### Lower genital tract infection and endometritis: insight on subclinical pelvic inflammatory disease

*Harold C. Wiesenfeld MD, CM, Sharon L. Hillier PhD, Marijane A. Krohn PhD, Antonio A. Amortegui MD, R. Phillip Heine MD, Daniel V. Landers MD and Richard L. Sweet MD*

*University of Pittsburgh, Magee-Women's Hospital*

**Objectives:** To investigate the association between lower genital tract infections (LGTI) and histologic endometritis among women without evidence of acute pelvic inflammatory disease (PID).

**Study design:** An observational study in which 556 women at risk for lower genital tract infections and without clinical evidence of acute PID were enrolled from STD and ambulatory clinics.

Vaginal samples were collected for Gram stain for bacterial vaginosis (BV) score, and cervical samples were obtained for culture for *Neisseria gonorrhoeae* and polymerase chain reaction (PCR) for *Chlamydia trachomatis*. Endometrial biopsies were processed for histologic analysis. Acute histologic endometritis was diagnosed in the presence of $\geq$ 5 neutrophils/400X and $\geq$ 1 plasma cell/120X of endometrial tissue, and plasma cell endometritis was defined as the presence of $\geq$ 1 plasma cell/120X field.

**Results:** Acute histologic endometritis was present in 74 women (13.3%), while 126 women (22.7%) had plasma cell endometritis and 356 women (64.0%) did not have endometritis. Acute histologic endometritis and plasma cell endometritis were more common among African-American women, women in the proliferative phase of the menstrual cycle, and among women who douched. Logistic regression analysis showed that BV, gonorrhea and chlamydia elevated the risk of acute but not plasma cell endometritis (Table 1).

**Conclusions:** Endometritis was common among women in the study with LGTI who did not have acute PID. Gonorrhea, chlamydia, and bacterial vaginosis were independently associated with acute histologic endometritis, but not plasma cell endometritis. These findings may represent sub-clinical PID. The reproductive sequelae of sub-clinical PID, such as infertility, ectopic pregnancy and chronic pelvic pain, remain to be characterized.

**Table 1** Association between endometrisis and vaginal bacteria

| | *Acute histologic endometritis* | | *Plasma cell endometritis* | |
|---|---|---|---|---|
| | *OR* | *95% CI* | *OR* | *95% CI* |
| Vaginal smear | 2.9 | 1.1–7.6 | 1.5 | 0.8–2.8 |
| Bacterial vaginosis (BV) | 2.0 | 0.6–6.1 | 1.3 | 0.6–2.8 |
| Intermediate flora | 2.7 | 1.0–7.0 | 1.5 | 0.8–2.7 |
| BV or intermediate flora | Ref | | Ref | |
| Normal flora | 2.7 | 1.3–5.8 | 1.8 | 0.9–3.6 |
| Gonorrhea (cervix) | 3.1 | 1.7–5.7 | 1.7 | 0.9–2.9 |
| Chlamydia (cervix) | | | | |

OR, odds ratio; CI, confidence interval

### Vaginal polymorphonuclear leukocytes and bacterial vaginosis as markers for acute endometritis

*M. H. Yudin MD, S. L. Hillier PhD, H. Wiesenfeld MD, CM, M. A. Krohn PhD, A. Amortegui MD and R. L. Sweet MD*

*University of Pittsburgh/Magee-Women's Hospital, Pittsburgh, PA*

**Objective:** To determine whether vaginal polymorphonuclear leukocytes (PMNs) predict acute endometritis among women without symptoms of pelvic inflammatory disease (PID).

**Study design:** Five hundred and forty-four women were enrolled from May 1998 to February 2001. They were recruited from four clinical sites. Inclusion criteria included age 15–30, infection with or risk factors for *Neisseria gonorrhoeae* (GC) or *Chlamydia trachomatis* (CT), or clinically diagnosed symptomatic bacterial vaginosis (BV). Vaginal Gram stains were evaluated for the presence of both BV and PMNs, and an endometrial biopsy was performed. Acute endometritis was diagnosed based on the presence of plasma cells and PMNs in the biopsy specimen. Statistical analysis was performed to determine whether there was an association between the presence of vaginal PMNs and acute endometritis, after stratifying for BV and sexually transmitted diseases (STDs).

**Results:** Of the 544 subjects enrolled 357 (65.6%) had BV, 11% had GC, and 21% had CT. Of those with BV, 158 (44.3%) had vaginal PMNs present, while 85 (45.4%) of those without BV had vaginal

PMNs present. Women with vaginal PMNs had a significantly higher prevalence of acute endometritis than those without vaginal PMNs (20% *vs.* 7%, $p < 0.001$). However, as shown below, women with both BV and vaginal PMNs had the highest risk of acute endometritis (Table 1).

Since PMNs could be related to concurrent STDs, women with GC or CT were excluded and the analysis was performed again; BV and PMNs remained associated with acute endometritis (OR 6.0; 95% CI, 1.2–40).

**Conclusions:** Vaginal PMNs are associated with acute endometritis, independent of BV or concurrent STDs.

**Table 1** Correlation of the incidence of acute endometritis with polymorphonuclear leukocytes and bacterial vaginosis

| | *Number of subjects* | *Acute endometritis* | *Odds ratio (OR)* | *95% Confidence interval* |
|---|---|---|---|---|
| BV+, PMN+ | 158 | 28 (17.7%) | 7.1 | (2.0–30) |
| BV-, PMN+ | 85 | 11 (12.9%) | 4.9 | (1.2–23) |
| BV+, PMN- | 199 | 14 (7.0%) | 2.5 | (0.7–11) |
| BV-, PMN- | 102 | 3 (2.9%) | Reference | Reference |

### Influences of vaginal and embryo transfer catheter microbiology on early pregnancy loss following *in vitro* fertilization

*L. O. Eckert MD, D. E. Moore MD, K. Agnew BS and D. A. Eschenbach MD*

**Objectives:** In women undergoing *in vitro* fertilization (IVF) bacterial vaginosis (BV) has been associated with increased early pregnancy loss, but no change in conception rate[1]. We conducted a pilot study of 91 women undergoing IVF to determine the influences of BV and IVF catheter tip cultures on conception and early pregnancy loss.

**Study design:** From May 1997 to May 1998, 91 women undergoing IVF at the University of Washington were enrolled in a pilot study to determine the effect of vaginal and catheter tip bacteria on live birth rate (LBR), and early pregnancy loss. All women had vaginal cultures and Gram stains (scored with the Nugent criteria), and embryo transfer catheter tips cultured. Early pregnancy loss was defined as a positive serum human chorionic gonadotropin (hCG) on day 14 after transfer, but no fetal heartbeat on ultrasound 6 weeks after transfer.

**Results:** LBR was 29 of 91 (31.9%), and early pregnancy loss was 14 of 42 (33.3%). The table below presents the conception rate and early pregnancy loss in women examined for BV and catheter tip cultures.

**Conclusions:** This small pilot study suggests that IVF patients with BV and catheter tip isolation of viridans streptococci may have decreased conception rates and increased early pregnancy loss. Those women with $H_2O_2$ + *Lactobacillus* on the catheter tip had the highest conception rate, and no early pregnancy loss. While this study is small, it suggests

**Table 1** Influences of vaginal and embryo transfer catheter microbiology on early pregnancy loss

| *Factor* | *+ hCG at day 14* | *Early pregnancy loss* |
|---|---|---|
| *Vaginal Gram stain* | | |
| BV flora (*n* = 8) | 2 (25.0%) | 1 of 20 (50.0%) |
| Int. flora (*n* = 33) | 13 (39.4%) | 5 of 13 (38.5%) |
| Normal flora (*n* = 50) | 27 (54.0%) | 8 of 27 (29.6%) |
| *Catheter tip culture* | | |
| Viridans streptococci (*n* = 17) | 3 (17.6%) | 2 of 3 (66.7%) |
| H2O2 + Lactobacillus (*n* = 10) | 7 (70.0%) | 0.0 |
| No bacteria (*n* = 57) | 18 (31.6%) | 6 of 18 (33.3%) |

that a larger prospective trial is warranted. Treatment of BV, viridans streptococci or other pathogens, and enhancement of $H_2O_2$ + *Lactobacillus* may lead to a higher IVF success rate.

### Reference

1. Ralph S, Rutherford A, Wilson J. Influence of bacterial vaginosis on conception and miscarriage in the first trimester: cohort study. *Br Med J* 1999; 319(7204):220–3

---

### The risk of acquisition of herpes simplex virus (HSV) during pregnancy: a prospective couples study

*Carolyn Gardella MD, Rhoda Ashley PhD, Sylvia Berry MS, Stacy Selke MS, Judy Zeh PhD, Lawrence Corey MD, Zane Brown MD*

*Departments of Obstetrics and Gynecology, Biostatistics, and Laboratory Medicine, University of Washington, Seattle, WA*

**Objective:** To determine the prevalence of herpes simplex virus (HSV) serologic discord between partners and the risk factors for transmission of HSV during pregnancy among HSV discordant couples.

**Study design:** Serum samples were obtained from women at the first prenatal visit, at the time of labor, and from their partners at any time during pregnancy or puerperium to test for HSV by Western blot. A woman was HSV-1 susceptible if she was HSV-1 seronegative and her partner was HSV-1 seropositive. Similarly, she was HSV-2 susceptible if she was HSV-2 seronegative and her partner was HSV-2 seropositive. The frequency of maternal seroconversion was calculated as the ratio of the number of women who had HSV-1 or -2 seroconversion during pregnancy to the number of women HSV-1 or HSV-2 susceptible.

**Results:** Of 4064 couples enrolled, maternal prenatal, delivery and partner sera were available from 3071 (75.6%) couples. Thirty-two percent of enrolled women were HSV seronegative, 46% were HSV-1, 10% were HSV-2, and 12% were HSV-1 and -2 seropositive. Thirty-five percent of the men were HSV seronegative, 49% were HSV-1, 8% were HSV-2, and 8% were HSV-1 and -2 seropositive. Of 3071 couples, 1457 (47.4%) were serologically discordant with 664 (21.6%) women being the susceptible partner. Among susceptible women, 540 (81.3%) were HSV-1 susceptible and 124 (18.7%) were HSV-2 susceptible. Of the HSV-1 susceptible women, 393 were HSV negative, of whom 13 (3.3%) seroconverted to HSV-1, and 147 were HSV-2 seropositive, of whom 1 (0.7%) seroconverted to HSV-1. Of the HSV-2 susceptible women, 32 were HSV negative, of whom 7 (21.9%) seroconverted to HSV-2, and 92 were HSV-1 seropositive, of whom 10 (10.9%) seroconverted to HSV-2.

**Conclusions:** The risk for seroconversion to HSV-2 was greatest (21.9%) among susceptible women who were HSV seronegative. Partner testing can identify women at high risk for primary HSV-2 infection during pregnancy for targeted safe-sex interventions.

**Acknowledgement:** This research was supported by NIAID grant AI-30731.

---

### A double-blind, randomized, placebo-controlled trial of acyclovir in late pregnancy for reduction of herpes simplex virus (HSV) shedding and cesarean section

*D. H. Watts, Z. A. Brown, D. Money, S. Selke, J. Aura, V. Remple, A. Cent, A. Hobson, S. Sacks, S. Berry and L. Corey*

*University of Washington and University of British Columbia*

**Objective:** To assess the efficacy of acyclovir compared to placebo in reducing the rate of genital herpes simplex virus (HSV) culture and polymerasae chain reaction (PCR) positivity and cesarean section among pregnant women with symptomatic genital HSV.

**Study design:** HIV-negative pregnant women with at least one symptomatic recurrence of genital HSV in the past year were randomized to acyclovir 400 mg TID or identical placebo from 36 weeks of

**Table I** Efficacy of acyclovir for the treatment of genital herpes simplex virus (HSV)

| Outcome | Acyclovir (n = 87) | Placebo (n = 83) | p |
|---|---|---|---|
| Any + genital culture | 10 (11.5%) | 33 (39.8%) | < 0.01 |
| Any + genital PCR | 30/84 (35.7%) | 45/75 (60.0%) | 0.01 |
| Days culture+ | 17/1833 (0.9%) | 72/1722 (4.2%) | < 0.01 |
| Days PCR+ | 96/1622 (5.9%) | 244/1317 (18.5%) | < 0.01 |
| Lesions at delivery | 4 (4.6%) | 12 (14.5%) | 0.04 |
| PCR+ at delivery | 1/28 (3.6%) | 6/24 (25.0%) | 0.04 |
| C-section for HSV | 3/87 (3.5%) | 9/83 (10.8%) | 0.08 |

gestation until delivery. The women had weekly visits, completed a daily symptom diary and obtained daily perineal and cervico-vaginal specimens at home for HSV culture and DNA detection by PCR. Obstetrical management was at the discretion of the attending physician; cesarean delivery for HSV was carried out if lesions were present. Analyses were done using chi-square and two-tailed Fisher's exact tests.

**Results:** There were no significant differences between treatment groups in demographic or obstetrical characteristics, or in the patients' history of herpes. Outcomes are summarized in the table.

Neonatal outcomes were similar between groups. No cases of neonatal HSV occurred.

**Conclusions:** Acyclovir, 400 mg TID orally, significantly reduced but did not eliminate HSV lesions and detection by culture and PCR in late pregnancy among women with symptomatic recurrent HSV. While C-section rates for HSV were reduced by suppressive therapy, we were not able to show a statistically significant reduction.

### Measurement of biogenic amines by ion mobility spectrometry (IMS) as an indication for bacterial vaginosis (BV)

*Chaim Walter, Silberstein Tali, Karpas Zeev, Lorber Avi, Katz Miriam and Mazor Moshe*

*Department of Obstetrics and Gynecology, Soroka University Medical Center Faculty of Health Sciences, Ben Gurion University of the Negev, Beer Sheva, and the Nuclear Research Center, Israel*

**Objective:** The presence of elevated levels of biogenic amines, such as putrescine, cadaverine and trimethylamine (TMA), in vaginal fluid is indicative of bacterial vaginosis (BV) and other pathological conditions. These are usually expressed in the Amsel test as a positive result in the 'Whiff test' and an increase in the pH of the sample to pH > 4.5. Ion mobility spectrometry (IMS) is an instrumental technique for identifying compounds and determining their concentrations, based on

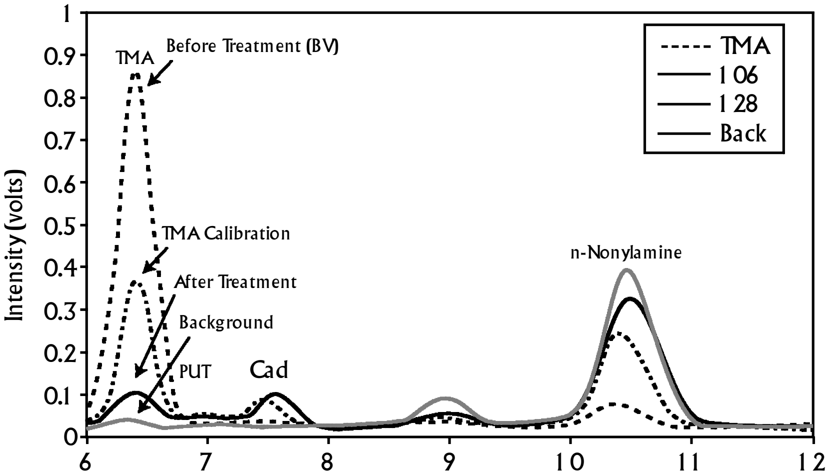

**Figure 1** Ion mobility spectrogram of vaginal fluid in bacterial vaginosis (BV). TMA, trimethylamine; Put, putrescine; Cad, cadaverine measurement of the velocity of ions (ion mobility) drifting through a bath gas (usually air at ambient pressure) under the influence of an electric field. The technique is particularly sensitive to amines.
**Study design:** A novel method for determining the biogenic amines content in a vaginal swab sample by use of IMS was developed. A few drops of an alkaline solution are added to the sample to enhance the emanation of the amines, and the mobility spectrum of the vapors entering the instrument is recorded. The analysis of a sample takes less than a minute.
**Results:** Elevated levels of TMA were found in vaginal fluid swabs taken from subjects diagnosed as having BV. Swabs taken during return visits, after treatment, clearly showed that the disappearance of the symptoms in the Amsel test were concordant with the return to normal TMA levels (Figure 1).
**Conclusion:** Preliminary work shows that ion mobility spectrometry has potential for diagnosis of other types of vaginitis.

---

### Congenital cytomegalovirus infection presenting as a molar pregnancy

*Christine Isaacs MD and Mara J. Dinsmoor MD*

*Department of Obstetrics and Gynecology, MCVH & P of the VCU Health System, Richmond, VA*

**Objective:** To report an unusual case of congenital cytomegalovirus (CMV) infection that presented with findings suggestive of a (partial) molar pregnancy.
**Description of case:** A 22-year-old G2P0A1, at 19 weeks and 6 days was referred with 'swollen feet and a headache'. Her medical history included a 'thyroid problem' for which she had been taking 'some medicine' until one month previously. Physical exam revealed blood pressure 135/74, P-80. She had no periorbital edema or thyromegaly. Her abdomen was tense and nontender and the fundal height was 23 cm with positive FHTs. 1+ lower extremity edema and 2+ deep tendon reflexes were present. Laboratory evaluation revealed 2+ urinary protein, β-hCG 502 306 mIU/ml, ast 199 units/l, ALT 175 units/l and thyroid stimulating hormone (TSH) 0.1 mIU/ml. Uric acid, ammonia, renal function, coagulation studies and platelets were normal. Ultrasound revealed a 19-week pregnancy with oligohydramnios and placentomegaly (14 cm in thickness entrapping the fetus), ascites and echogenic bowel. The heart appeared to be enlarged but structurally normal. The patient elected to undergo termination. We suspected that this constellation of findings represented a partial mole with associated pre-eclampsia and thyrotoxicosis. Findings at D & E included a large amount of placental tissue with hydropic villi. Postoperatively, the LFTs quickly returned to normal levels. Serologic evaluation ultimately revealed positive CMV IgM and IgG. Pathologic examination of the placenta revealed no histologic evidence of a molar pregnancy. Eosinophilic cytoplasmic and nuclear inclusions, consistent with CMV infection, were noted. Further immunohistochemical staining confirmed the presence of placental CMV infection.
**Conclusion:** Congenital CMV infection may present in an unusual manner. The practitioner must maintain a high index of suspicion for congenital infection.

### Direct comparison of Abbott LCX and GenProbe Pace II for gonorrhea and chlamydia

*Jeanna M. Piper MD, William A. Peairs MA, Rochelle N. Shain PhD, Sondra Perdue DrPH and Jane D. Champion PhD*
*Department of Obstetrics and Gynecology, UTHSC-SA, San Antonio, TX*

**Objectives:** To compare the sensitivity and specificity of Abbott LCX with those of GenProbe Pace II for the detection of cervical infection with *Neisseria gonorrhea* (GC) or *Chlamydia trachomatis* (CT) in high-risk women.
**Study design:** 265 consecutive women undergoing testing by cervical swab for GC and CT using the GenProbe Pace II (collected first) had an additional cervical swab obtained for performance of the Abbott LCX (collected after the GenProbe sample). Both assays were run according to the manufacturer's standard protocols.
**Results:** Thirty-four women had a positive test for CT (12.8%) and 15 women had a positive test for GC (5.7%). CT was detected by LCX in 31/34 (91.2%) and by GenProbe in 24/34 (70.6%) with concordance noted in 21 cases (61.8%), only LCX+ in 10 (29.4%) and only GenProbe+ in 3 (8.8%). GC was detected by LCX in 14/15 (93.3%) and by GenProbe in 14/15 (93.3%) with concordance noted in 13 cases (86.7%), only LCX+ in 1 (6.7%) and only GenProbe+ in 1 (6.7%). Six women had a positive test for both GC and CT with 3 cases concordant, 1 GC+ by GenProbe only, 1 CT+ by GenProbe only and 1 CT+ by LCX only.
**Conclusions:** The use of LCX technology using cervical samples increased the detection of CT by 30% in a cohort of high-risk women, but did not impact on the detection of GC in the same population. Increased use of DNA amplification techniques in clinical practice will result in improved detection and potentially reduced morbidity owing to the opportunity for administering earlier therapy for cervical infections caused by CT.

---

### Intracellular interleukin-4 (IL-4) cytokine production in HIV+ and HIV- women

*Arlene Bardeguez MD, Bart Holland PhD, Thomas Denny BS, Madeline Sutton MD, Zeneida Garcia BS, Janet Stein BS and Paul Palumbo MD*
*New Jersey Medical School, Newark, NJ*

**Objective:** To evaluate intracellular **i**nterleukin-4 (Il-4) production in CD4 and CD8 lymphocytes among HIV-infected pregnant women and seronegative counterparts.
**Study design:** Sixty-one women were enrolled in the study (31 HIV+, 30 HIV-) and after obtaining informed consent study visits were scheduled every trimester and postpartum for the pregnant women, while the non-pregnant women had only one evaluation. HIV-infected women were staged based on the adult adolescent CDCP classification. At each study visit participants had peripheral whole blood drawn to analyze for (1) lymphocyte profile, (2) measurement of intracellular cytokine production and (3) viral load. Medical records were reviewed to abstract information regarding HIV medications and medical/obstetrical complications. Fischer's exact test, and Rank sums were used for analysis.
**Results:** HIV-infected women were more likely to be older (28 ± 7 yrs *vs*. 24 ± 5 yrs; $p = 0.04$) and have a past or present history of substance use ($p = 0.0001$). There were significantly lower CD4 counts and higher CD8 counts among HIV-

**Table 1** Levels of Cd4 and Cd8 cells, and interleukin-4 (IL-4) in them

| | *HIV+* | *HIV-* | *p* |
|---|---|---|---|
| *Time 0* | | | |
| Cd4 | 463 ± 270 | 714 ± 262 | 0.04 |
| Cd4-IL4 | 6.5 ± 2.1 | 4.3 ± 1.8 | 0.030 |
| Cd8 | 913 ± 349 | 436 ± 124 | 0.0005 |
| Cd8-IL4 | 4.8 ± 2.7 | 4.3 ± 1.2 | 0.82 |
| *Time 3* | | | |
| Cd4 | 482 ± 239 | 892 ± 262 | 0.002 |
| Cd4-IL4 | 6.7 ± 2.8 | 4.7 ± 2.9 | 0.029 |
| Cd8 | 1141 ± 919 | 617 ± 235 | 0.01 |
| Cd8-IL4 | 3.2 ± 1.9 | 3.0 ± 2.2 | 0.80 |

infected women compared to seronegative counterparts (Table1). IL-4 levels in CD4 cells were higher among HIV-infected women during pregnancy and postpartum but production of IL-4 in CD8 cells was no different between HIV-positive and negative women. Intracellular IL-4 production was correlated with disease stage, CD8 counts and viral load.

**Conclusion:** Intracellular IL-4 production was primarily observed only within CD4 cells in our cohort. There was a higher production of IL-4 among HIV-infected women through pregnancy and postpartum. Pregnancy could further enhance the balance toward a TH-2 response elicited by HIV infection.

---

### Vaginitis due to *Candida krusei*: epidemiology, clinical aspects, and therapy

*Shivani Singh MD, Jack D. Sobel MD, Pallavi Bhargava MD and Jose Vazquez MD*

**Objectives:** To study the epidemiology, clinical aspects, and therapy of *C. krusei* vaginitis.
**Study design:** Retrospective chart review of 10 women with *C. krusei* in 25 vaginal samples over the last 15 years with MIC testing and molecular typing.
**Results:** The ages of the women in the study group ranged from 32 to 63 years, with a mean of 44 years; eight of the ten were Caucasian. Seven of the women were pre-menopausal, of whom four had had previous bacterial vaginosis. The patients frequently had chronic, refractory vulvovaginal signs and symptoms, which were otherwise indistinguishable from vaginitis due to other yeasts. These patients had received multiple antimycotics including fluconazole and miconazole. On wet films eight of the ten showed budding yeast only and two had yeast and hyphae. Three of the ten had a second consecutive yeast species while two had a coexistent yeast species. The MIC90 of fluconazole was > 64 mg/ml, that of miconazole was 4 mg/ml; the most active azole was clotrimazole, with a MIC90 of 0.25 mg/ml. Three out of four patients treated with clotrimazole and four out of five treated with boric acid responded clinically and mycologically. Two dominant karyotypes were identified on CHEF; no major karyotype change was seen in successive samples of the same patient, confirming vaginal relapse in these refractory cases.
**Conclusions:** *C. krusei* is a rare but important cause of vaginitis in women over 30 years of age. It is highly resistant to fluconazole and miconazole. The recommended treatment is prolonged therapy with clotrimazole or boric acid.

### References

1. Sobel *et al.* Vaginitis due to *Saccharomyces cerevisiae*: epidemiology, clinical aspects, and therapy. *CID* 1993;16:93–9
2. Vazquez *et al.* Comparison of REA vs. pulsed-field gradient gel electrophoresis as a typing system for *Torulopsis glabrata* and *Candida* species other than *C. albicans*. *J Clin Microbiol* 1993;8:2021–30

---

### The effect of temperature on the bactericidal properties of 10% povidone-iodine solution

*Michael P. Leung MD, Karen D. Bishop BS and Manju Monga MD*

*Department of Obstetrics, Gynecology and Reproductive Sciences, University of Texas Houston Medical School, Houston, TX*

**Objectives:** Ten percent povidone-iodine (PVI) is commonly used as a bactericidal solution prior to amniocentesis; the use of warm PVI may increase patient comfort. However, manufacturers suggest that PVI be stored at room temperature, and the effect of warming on its bactericidal properties is not known. The objective of this study was to determine the effect of warming on the bactericidal properties of 10% PVI.
**Methods:** *In vitro* experiments were conducted in 25°C (room temperature) and 32°C water baths, the latter temperature being maintained by the use of an ultrasonic gel warmer. Nine ml of PVI was dispensed into sterile culture tubes at each temperature and 1 ml of bacteria ($10^7$ organisms/ml *S. aureus, Enterococcus, E.coli*, group B *Streptococcus*) was added. After 0.25, 0.5, 1, 2, 4 and 8 minutes, 1 ml samples were removed and added to 4 ml 0.5% sodium thiosulfate (to neutralize the iodine and interrupt bactericidal action). The number of viable organisms was determined by plating 0.1 ml samples on TSA plates. Plates were incubated at $37^0$C for 24 hours and CFUs counted. For *in vivo* experiments in 20 volunteers, a 9 $cm^2$ area of the dorsum of each hand was cultured and then wiped for 15 seconds with PVI at 25°C or 32°C. Each hand was re-cultured using TSA plates (after 48 hour culture). The ability to kill more than 99% of organisms after 15 seconds using PVI at each temperature was compared. The Mann–Whitney U test was used where appropriate and a $p$ value $< 0.05$ was considered significant.

**Results:** *In vitro*, PVI was bactericidal against *E. coli, S. aureus, Enterococcus* and group B *Streptococcus* within 0.25 minutes at both 25°C and 32°C with median bacterial growth of no CFU/plate for each bacterium studied (three replicates at each temperature). Median bacterial growth from skin was 6 CFU/plate (range 0 to more than 100). After wiping with PVI at 25°C and 32°C, median bacterial growth was 1 CFU/plate (range 0–3) and 0 CFU/plate (range 0–4), respectively (NS).

**Conclusion:** PVI is as effective at 32°C as at 25°C. The use of PVI at 32°C should be considered in procedures performed without anesthetic.

---

### Cellulose sulfate inhibits *Gardnerella vaginalis* and other anaerobes associated with bacterial vaginosis

*Jose A. Simoes[1,2], Lourens J. D. Zaneveld[1], Alla Aroutcheva[1], Calvin J. Chany II[1], Robert A. Anderson Jr[1], Donald P. Waller[3] and Sebastian Faro[4]*

*[1]Department of Obstetrics and Gynecology, Rush Medical College, Chicago, IL; [2]Department of Obstetrics and Gynecology, State University of Campinas, SP, Brazil; [3]Department of Pharmaceutics and Pharmacodynamics, College of Pharmacy, University of Illinois at Chicago, Chicago, IL; [4]Department of Obstetrics and Gynecology, The University of Texas Health Science Center-Houston, and The Woman's Hospital of Texas, Houston, TX*

**Introduction:** A high molecular weight sulfated polysaccharide (approximately 1900 kDa) cellulose sulfate (H-CS) is a new vaginal antimicrobial contraceptive compound that can inhibit the replication of human immunodeficiency virus type 1, herpes simplex virus, and the growth of *Neisseria gonorrhoeae* and *Chlamydia trachomatis* but not the growth of lactobacilli. It is important that vaginal microbicides do not alter the endogenous vaginal microflora resulting in conditions such as bacterial vaginosis (BV).

**Objectives:** To evaluate whether H-CS can inhibit the *in-vitro* growth of *Gardnerella vaginalis* and other anaerobic bacteria associated with BV.

**Study design:** Eight clinical isolates from patients with BV were used for this preliminary study, including strains of *G. vaginalis, Prevotella bivia, Peptostreptococcus asaccharolyticus* and *Peptostreptococcus anaerobius*. Samples of H-CS were serially diluted in a range between 10 mg/ml and 0.125 mg/ml. The macrodilution broth method was performed according to National Committee for Clinical Laboratories Standards to evaluate the effect of H-CS on the growth of anaerobic bacteria. The zone of growth-inhibition in HBT agar plate was used to evaluate its effect on *G. vaginalis* growth.

**Results:** A dose-dependent growth-inhibition effect of H-CS was found against all *G. vaginalis,* as well as *P. bivia*, *P. asaccharolyticus* and *P. anaerobius* clinical isolates.

**Conclusions:** These preliminary results suggest that vaginally applied H-CS may inhibit the growth of BV-associated organisms and support the use of H-CS as a safe vaginal microbicide. Further studies need to be performed to confirm its clinical efficacy.

### HPV subtypes, vulvar dysplasia and HIV-infection

*Deborah Cohan MD, Abner Korn MD and Joel Palefsky MD*

**Objectives:** To investigate the role of HPV and determine the HPV subtypes involved in vulvar intraepithelial neoplasia (VIN) among HIV-infected and uninfected women.

**Study design:** A case-control study was performed among HIV-positive and HIV-negative women diagnosed with VIN. Medical charts were abstracted for age, tobacco use, and primary *vs.* recurrent VIN. The specimens were graded as VIN 1, VIN 2, or VIN 3, utilizing standard criteria. Polymerase chain reaction (PCR) was performed on DNA preparations from stored vulvar specimens using consensus HPV L1 primers. Specimens were then probed for 39 different HPV subtypes. Variables were analyzed using the $\chi^2$ test, Fisher's exact test, or *t* test, as appropriate, using EpiInfo2000 (CDC, Atlanta, GA).

**Results:** Thirty-two HIV-positive cases and 20 HIV-negative controls were included in the study. There were no significant differences in age, primary *vs.* recurrent nature of VIN, VIN grading or tobacco use between cases and controls. There was no difference in the prevalence of multiple HPV types or unusual HPV types in cases (9%) versus controls (10%). 63% of HIV-positive women with VIN had no detectable HPV as compared to 40% of HIV-negative women.

**Conclusions:** There was very low recovery of HPV overall, but particularly in HIV-infected women. This may be owing to unique HPV types not found on the available probes. Our finding of 3 cases among HIV-infected women in which HPV was present but could not be further sub-typed supports this hypothesis. On the other hand, VIN could be caused by an etiology other than HPV in HIV-infected women. Further studies are necessary to investigate the natural history of HPV in HIV-infected women and to examine possible atypical HPV types and co-factors in the development of vulvar dysplasia.

---

### Phage infection of lactobacilli: prevalence and host ranges

*L. Tao[1], S. I. Pavlova[1], A. O. Kiliç[1,2], V. S. Ocana[3], M. E. Nader-Macías[3], J. A. Simoes[4], E. E. Castro[5] and J. S. So[6]*

*[1]University of Illinois at Chicago, USA; [2]Karadeniz Technical University, Trabzon, Turkey; [3]CERELA, CONICET, Argentina; [4]State University of Campinas, Brazil; [5]University of Concepcion, Chile; [6]Inha University, S. Korea*

**Objectives:** Since a reduction of vaginal lactobacilli is associated with bacterial vaginosis, it is important to study phages that infect lactobacilli. The aims of this study were to determine the species of phages and lactobacilli in the human vagina, the prevalence of phage infection in different populations, and the host ranges of these infective phages.

**Study design:** Vaginal samples were collected from reproductive-aged women in Turkey and the USA to determine phage types. Vaginal samples from women of other countries, including Brazil, Argentina, Chile, China, India and S. Korea, were analyzed for *Lactobacillus* species and for establishing a phylogenetic tree based on their 16S rRNA sequences. Finally, chromosomal DNAs from these lactobacilli were probed with DIG-labeled genomic DNA from two representative type phages, kc5a and TL34, to determine the prevalence of phage infection and the host ranges.

**Results:** Sixty-seven phages were isolated from the vaginal lactobacilli of 209 women in the USA and Turkey, among which two major phage types were identified. Out of 450 vaginal lactobacilli isolated from women from different countries, 35 representative strains were selected for species determination. Their 16S rRNA gene sequences were used to establish a phylogenetic tree. Most lactobacilli belonged to three major species, *L. crispatus, L. jensenii,* and *L. gasseri*. Minor species included *L. fermentum, L. vaginalis, L. mucosae, L. paracasei*, *L. rhaminosus*, and two new phylotypes. None of the vaginal *Lactobacillus* species were the same as those found in food. Phage infection was not limited to specific *Lactobacillus* species. Lactobacilli infected by phages were common among women in the cities of the USA, Turkey, Brazil and Chile, but rare among women in South Korea and northern Argentina.

**Conclusions:** Two major phage types and multiple *Lactobacillus* species were identified in the vagina of women. Phage infection of lactobacilli did not appear to be species specific. The prevalence of phage infections varied significantly among women in different areas.

---

### Ampicillin-resistant *Escherichia coli* in gestational pyelonephritis: increased occurrence and association with the colonization factor DR adhesin

*Audrey Hart BA*[1], *Bogdan J. Nowicki MD, PhD*[1,2], *Barbara Reisner*[3], *Edyta Pawelczyk*[1], *Pawel Goluszko MD, PhD*[1], *Petri Urvil PhD*[1], *Garland Anderson MD*[1] *and Stella Nowicki DDS*[1,2]

[1]*Division of Infectious Diseases, Department of Obstetrics & Gynecology,* [2]*Department of Microbiology & Immunology and* [3] *Department of Pathology, The University of Texas Medical Branch at Galveston*

**Summary:** We evaluated the pattern of ampicillin resistance and possible association with virulence factors of 78 *Escherichia coli* isolates from 78 pregnant women with pyelonephritis. The occurrence of ampicillin resistance was significantly higher among pyelonephritis isolates (46%) than that reported in 1985 (22%). Resistance was found more frequently during the first (60%) and third (53%) trimester than during the second (33%) trimester. Of all *dra*+ *E. coli* isolates, 75% were ampicillin-resistant, while *dra*+ of O75 serotype *E. coli* accounted for 87% of ampicillin-resistant strains. The significant increase of ampicillin resistance among gestational pyelonephritis *E. coli* and association with the *dra* gene cluster encoding colonization and invasive capacity may warrant further study involving obstetrics and neonatal wards, with the last being at the highest risk for potential problems.

---

### Serum complement activity during menses as a risk factor for gonococcal pelvic inflammatory disease

*Audrey Hart BA*[1], *Petri Urvil PhD*[1], *Bodgan Nowicki MD, PhD*[1,2] *and Stella Nowicki, DDS*[1,2]

[1]*Department of Obstetrics and Gynecology, and* [2]*Department of Microbiology and Immunology, The University of Texas Medical Branch at Galveston, Texas, 77555-1062*

**Objective:** Symptoms of gonococcal pelvic inflammatory disease usually occur at the onset of menses. Our recent study showed that the bactericidal activity of normal human serum (NHS) decreased during menses. Complement C1q plays a crucial role in both bactericidal activity and gonococcal virulence. Therefore we evaluated the serum complement C1q level and C1q binding to *Neisseria gonorrhoeae* and compared them to complement activity pre-, during and post-menses.

**Design:** Serum from four female subjects of reproductive age without a prior history of gonococcal infection was obtained from blood drawn pre-, during and post-menses. Serum C1q level and complement activity were measured throughout the menstrual cycle. The interaction of C1q with *N. gonorrhoeae* cells was analyzed in immunoblots.

**Results:** The results indicated that complement activity decreased during menses, corresponding to reduced bactericidal activity of the serum. Complement and bactericidal activity were inversely related to serum levels of C1q and C1q interaction with *N. gonorrhoeae*.

**Conclusion:** Decreased complement activity may account for the reduced bactericidal activity of serum during menses, while increased C1q interaction with *N. gonorrhoeae* may enhance gonococcal virulence during the onset of menses. These results also suggest that complement activity and complement components may be regulated by sex hormones. Decreased complement activity and increased C1q binding combined may predispose women to a higher risk of developing gonococcal pelvic inflammatory disease during menses.

### Placental histopathology of congenital syphilis correlated to pregnancy outcome

*Jeanne S. Sheffield MD, David W. I. Fong MD, Pablo J. Sanchez MD, George D. Wendel Jr MD, Linda R. Margraf MD, Donald D. McIntire PhD and Beverly Barton Rogers MD*

*Departments of Obstetrics and Gynecology, and Pediatrics and Pathology, University of Texas Southwestern Medical Center, Dallas, TX*

**Objective:** To evaluate the contribution of placental histopathology to the diagnosis of congenital syphilis.
**Study design:** The study was a retrospective cohort analysis from January 1 1986 to December 31 1998. Women who delivered with a reactive serology for syphilis and who had received no prior treatment were evaluated and clinically staged. Infants were examined and underwent long-bone radiographs and laboratory testing. In addition, infants had specific IgM immunoblotting, polymerase chain reaction (PCR) and/or rabbit infectivity testing. Placental histopathology was performed and the findings correlated with the outcome of pregnancy.
**Results:** Sixty-seven women admitted for delivery had reactive syphilis serologic testing and placentas available for review. There were 33 (49.3%) stillborn infants and 18 (26.9%) liveborn infants with congenital syphilis, 15 (22.4%) uninfected liveborn infants and one uninfected stillborn fetus. There were no differences between the groups with regard to demographic characteristics, prenatal care or stage of syphilis. Stillborn infants were more likely to delivery preterm ($p < 0.001$). Controlling for gestational age, histopathology revealed necrotizing funisitis, villous enlargement and acute villitis associated with congenital syphilis. Erythroblastosis was more common in stillborn infants with congenital syphilis than liveborn infants with or without congenital syphilis (OR 16; 1.6–69). Using physical exam, laboratory analysis and placental histology, 89% and 97% of liveborn and stillborn infants with congenital syphilis, respectively, were detected compared with 67% and 94% using physical exam and laboratory analysis alone.
**Conclusions:** Histopathologic examination of the placenta is a valuable adjunct to the standard criteria used to diagnose congenital syphilis.

### Efficacy of common antiretroviral regimens during pregnancy

*James B. Hill MD, Jeanne S. Sheffield MD and George D. Wendel Jr MD*

*Department of Obstetrics and Gynecology, University of Texas Southwestern Medical Center, Dallas, TX*

**Objective:** To compare the efficacy of common antiretroviral regimens in pregnant women in decreasing the maternal viral burden.
**Methods:** A retrospective chart review of HIV-infected women was performed and analyzed with respect to treatment regimen between May 1997 and January 2001. Plasma HIV-1 viral burden and CD4 count were analyzed at the first prenatal visit and at delivery. The interval from initiation of medication to delivery, infant outcomes and mode of delivery were noted and statistical analysis was performed using the ANOVA and Kruskal-Walis test.
**Results:** Study criteria were met and results were available for 121 women over the 3.5-year time period. Thirty-one women (25.6%) received combivir alone, 77 (63.6%) received combivir and a protease inhibitor (PI), and 13 (10.7%) received combivir and a non-nucleoside reverse transcriptase inhibitor (NNRTI). Table 1 lists the outcomes of interest in the 3 treatment regimens.

**Table 1** Viral load at baseline and delivery

| | *Combivir (n = 33)* | *Combivir + PI (n = 77)* | *Combivir + NNRTI (n = 13)* | *p value* |
|---|---|---|---|---|
| *HIV-1 viral load (copies/ml)* | | | | |
| Baseline, median (range) | 663 (< 400–56 451) | 5175 (< 400–173 000) | 440 (< 400–28 458) | NS |
| Delivery, median (range) | < 400 (< 400–23 302) | < 400 (< 400–48 797) | < 400 (< 400–2334) | NS |
| < 1000 copies/ml (%) | 26 (78.8) | 65 (84.4) | 11 (84.6) | NS |
| < 400 copies/ml (%) | 23 (69.7) | 52 (67.5) | 10 (76.9) | NS |
| CD4 count (per $mm^3$) | | | | |
| Baseline, mean $\pm$ SD | $515 \pm 265$ | $410 \pm 227$ | $527 \pm 225$ | 0.005 |
| Delivery, mean $\pm$ SD | $622 \pm 344$ | $496 \pm 245$ | $617 \pm 263$ | NS |
| Interval from baseline to delivery (days) | $210 \pm 194$ | $160 \pm 76$ | $156 \pm 57$ | NS |

Five patients underwent cesarean delivery for high viral loads; no infants in this cohort developed HIV infection

**Conclusion:** Combination antiretroviral therapy reduces plasma HIV-1 viral burden to less than 400 copies/ml in approximately 80% of HIV-infected pregnant women. Utilizing an aggressive anti-retroviral regimen reduces viral loads, and potentially decreases the rate of vertical HIV-1 transmission, allowing most women to undergo a vaginal delivery.

### Evaluation of clinical methods for diagnosing bacterial vaginosis

*Robert E. Gutman MD, Jeffrey Blume PhD, Walter Heber MS, Jeffrey F. Peipert MD, MPH*

*Women & Infant's Hospital, Brown University Medical School, Providence, RI*

**Objectives:** Current clinical criteria for the diagnosis of bacterial vaginosis (BV) include (1) a thin, homogeneous discharge, (2) pH > 4.5, (3) the release of amine odor with the addition of base, and (4) the presence of clue cells on microscopic evaluation of saline wet prep. A positive clinical diagnosis is made when three or more criteria are present. The objective of this study was to determine if the clinical criteria can be simplified without significant loss of diagnostic sensitivity or specificity. More specifically, we believe that the presence of two criteria may be as accurate as the presence of three or more of the clinical criteria

**Study design:** The study was a cross sectional analysis of fifty-nine subjects enrolled in clinical research studies and women who were undergoing a vaginal exam in the primary care clinic. All four clinical criteria were collected as well as the FemExam card. A Gram stain was performed for a definitive diagnosis. Calculations of sensitivity, specificity and their respective 95% confidence intervals (CI) are presented for each individual criterion and combinations of clinical criteria (Table 1).

**Table 1** Sensitivity and specificity of the criteria for BV, alone or combined ($n$ =59; 95% CI in brackets)

| | *Sensitivity* | *Specificity* |
|---|---|---|
| Vaginal discharge | 0.76 (0.65–0.89) | 0.53 (0.40–0.66) |
| pH > 4.5 | 0.86 (0.77–0.95) | 0.81 (0.71–0.91) |
| Positive amine odor | 0.77 (0.66–0.88) | 0.95 (0.89–1.00) |
| Clue cells present (> 20%) | 0.73 (0.62–0.84) | 0.92 (0.85–0.99) |
| Elevated pH and amine odor | 0.73 (0.62–0.84) | 0.97 (0.93–1.00) |
| Elevated pH and clue cells | 0.68 (0.56–0.80) | 0.97 (0.93–1.00) |
| Clue cells and amine odor | 0.68 (0.56–0.80) | 0.97 (0.93–1.00) |
| Amsel's criteria ($\geq$ 3 of 4 criteria) | 0.76 (0.65–0.87) | 0.97 (0.93–1.00) |

**Results**: The results are presented in Table 1.
**Conclusions:** From this initial analysis we believe that the combination of two clinical criteria can perform as well as 3 or more (Amsel's) criteria. After further recruitment, ROC analysis will be performed to confirm our preliminary findings.

---

### Cervical manipulation and membrane 'stripping' associated with stillbirth caused by group B Streptococcus and other perinatal pathogens: cervical membrane disruption syndrome (CMDS)

*C. A. Stamm MD, L. A. Bishop BA, J. A. McGregor MD, CM, J. G. McFee MD and M. Perach*

*Denver Health Medical Center, University of Colorado School of Medicine, Denver, Colorado, and Pasadena, California*

**Objective:** We present five cases in which cervical manipulation ('membrane stripping') intended to facilitate labor preceded perinatal sepsis and stillbirth caused by invasive Group B *Streptococcus* (GBS) as well as other perinatal pathogens.
**Study design:** Each case presented with similar salient features – term gestation in a previously healthy pregnancy, elective or non-urgently indicated promotion of labor and digital cervical manipulation.
**Results:** In each case there was rapid labor with placental findings of histologically severe intra-uterine infection or funisitis, often in the absence of classical clinical criteria of chorioamnionitis (CAM). One woman suffered observed fetal death due to overwhelming fetal GBS sepsis 15 hours after 'membrane stripping' to induce labor at term.
**Conclusion:** These cases support prior evidence that cervical manipulation is associated with translocation of vaginal microbes into the lower uterus[1]. The incidence of such presumably rare occurrences can only be established in large prospective epidemiologic studies. We suggest that our case definition be utilized in future studies. Clinicians may reconsider elective cervical manipulation in patients with cervical vaginal infection or colonization with potential perinatal pathogens, including GBS, and should identify and treat cervical infection or consider a GBS-specific chemprophylaxis prior to membrane stripping.

### Reference

1. McGregor, Farkouh. *IDSOG* 1998;89

---

### Apoptosis in the chorion laeve of term patients with histologic chorioamnionitis

*A. P. Murtha[1], V. D. Dew[1] and R. Bentley[2]*

*Departments of Obstetrics and Gynecology[1] and Pathology[2], Duke University Medical Center, Durham, NC*

**Objective:** In a pilot study of term fetal membranes from surgical pathology specimens, we previously reported increased apoptosis in the chorion laeve of patients with histologic chorioamnionitis. The objective of this investigation was to confirm our previous results by determining the extent of apoptosis in the chorion laeve in term patients with and without histologic chorioamnionitis.
**Study design:** 134 fetal membrane rolls were collected immediately after delivery remote from the rupture site and within 2 cm of the edge of the

**Table 1** Number of nuclei and apoptotic nuclei in chorion laeve in patients with and without chorioamnionitis

| | *Chorioamnionitis* (n = 17) | *No chorioamnionitis* (n = 17) | *p value* |
|---|---|---|---|
| Total nuclei, median (range) | 520 (323–734) | 543 (378–735) | NS |
| Total apoptotic nuclei, median (range) | 21 (3–68) | 9 (1–56) | $p = 0.0004$ |
| Percent positive nuclei, median (range) | 4.3 (0.5–9.3) | 1.7 (0.2–1.5) | $p < 0.0001$ |

placenta. Samples were fixed in formalin, embedded in paraffin and examined for evidence of histologic chorioamnionitis by a single pathologist. All membrane rolls were stained for apoptosis using the TUNEL method (Oncor, Gaithersburg, MD). The extent of apoptosis was quantified by counting the number of apoptotic nuclei in the chorion laeve relative to the total number of nuclei in 5 randomly chosen high-powered fields. Samples were divided into those with and without histologic chorioamnionitis. Clinical and demographic data were collected by chart abstraction and data were analyzed by the Mann–Whitney U test with significance defined as $p < 0.05$.

**Results:** There were no significant differences in maternal age, race, insurance status, cesarean delivery or gestational age at delivery between the two groups. Chorion laeve of fetal membranes from term patients with histologic chorioamnionitis had significantly more apoptotic nuclei than those without chorioamnionitis (Table 1).

**Conclusions:** These data confirm the finding of our pilot investigation that apoptosis is accelerated in the chorion laeve of term subjects with histologic chorioamnionitis. Intra-amniotic infection or inflammation may be associated with accelerated cell death in this important cell layer. Additional studies are needed to determine the clinical significance of this finding.

---

### Primary cultures of endocervix and ectocervix demonstrate unique cytokine secretion patterns

*Michael A. Nicolazzo BS, Jean J. Latimer PhD, Crystal M. Kelly BS and Deborah L. Draper PhD*

*Magee Women's Research Institute, Department of Obstetrics and Gynecology School of Medicine, University of Pittsburgh, Pittsburgh, PA 15213*

**Objective:** The study of early epithelial cytokine responses during infection is an emerging field of mucosal immunology. The endocervix has not been successfully studied and traditional models of female genital tract infections have relied on transformed or immortalized cells in tissue culture[1]. The aim of this study was to design an *in vitro* endocervical primary tissue culture model which maintains the relevant structures, epithelial polarity, extracellular matrix contact and longevity, and which can be used for comparison with ectocervical cultures for early cytokine responses.

**Study design:** Fresh tissue (1–2 $cm^2$) from the endocervix and ectocervix was taken from premenapausal women undergoing hysterectomy ($n = 4$) for non-infectious or non-cancer causes. Tissues were processed using the Latimer method (patent pending[2]). Cells were cultured with the novel MWRI epithelium medium and established on extracellular matrix (Matrigel). Digital images of growth patterns and cellular morphology were captured every 2–4 days and correlated to secreted levels of IL-1β, IL-6, IL-8, IL-10 and SLPI as measured by enzyme linked immunosorbent assay (ELISA).

**Results:** Endocervical epithelium grew well, could be sub-cultured for at least 4 passages, and formed glandular-like epithelial structures with connecting channels on a layer of fibroblasts (about days 14–21). Ectocervix formed typical multilayered epithelial cells on fibroblasts (days 15–18). Cytokine release patterns were initially modulated and then stabilized out to 120 days in culture. Comparisons of cytokine levels in endocervical with those in ectocervical culture supernatants demonstrated unique secretion patterns with significant differences ($p < 0.05$).

**Conclusions:** Primary genital epithelial cultures of endocervix and ectocervix can be established with the MWRI method and can be used for the measurement of early, relevant cytokine response patterns.

### References

1. Fichorova, Anderson. *Biol Reprod* 1999;6: 508–14
2. Latimer *et al.* 2000; U.S. Patent # 6074874

### Neurologic impairment among offspring of mothers with an intrauterine device (IUD) during pregnancy: is there an 'IUD baby syndrome'?

*J. A. McGregor, C. Stamm, K. Davis and J. E. Berg*

*Denver Health Medical Center, Denver, CO; New York, NY; and University of Colorado School of Medicine, Denver, CO*

**Objective:** The aim of this study was to describe and analyze persistent neurologic impairment(s) among five adult offspring conceived and gestated with an intrauterine device (IUD) in place during pregnancy. Perinatal white matter damage (WMD) associated with intrauterine inflammation is hypothesized to be a possible intermediate factor between the presence of an IUD during gestation and persistent neurologic/neurobehavioral disorders[1].
**Study design:** We analyzed developmental findings and neurologic assessments in a convenience sample of five adults who had been exposed to an IUD during their gestation.
**Results:** The subjects were a convenience sample of young adults exposed to an IUD during gestation, all of whom were between 26 and 29 years of age; four were female. Each subject was associated with the presence of a Dalkon Shield which could not be removed or was intentionally left in place during pregnancy. Preterm birth and premature rupture of membranes were common (three out of the five cases) as was maternal febrile morbidity/sepsis (two out of five). Spastic cerebral palsy (CP) occurred in two of the five. Developmental delay and academic failure were present in all cases as were deficiencies in spatial orientation. Clinical diagnosis and treatment of severe depression (3/5), anorexia (2/4 females), and obsessive-compulsive disorder (2/5) were common. The subjects were consistently described as lacking 'common sense', socially isolated and 'clumsy'. Only one suffered seizures.
**Conclusion:** We describe five individuals demonstrating persistent, neurologic and/or neurobehavioral abnormalities during childhood and adulthood who were exposed to a Dalkon Shield IUD during gestation. Further studies are required to confirm these putative associations of abnormalities with the presence of an IUD during gestation and discover possible mechanisms for them. The possible existence of a so-called 'IUD baby syndrome' may prompt care providers to ensure the removal of an IUD during gestation or, alternatively, to scrupulously identify and treat reproductive tract infections during pregnancy with a retained IUD.

### Reference

1. Damman O, *et al. Curr Opin Pediatr* 2000;12: 99–104

---

### Imiquimod 5% cream is safe and effective in female patients with external genital warts: Results of an open-label multi-center trial

*Michel Fortier, MD*

*University of Laval, Quebec City, Quebec*

**Objective:** To determine the safety and efficacy of imiquimod 5% cream, an immune response modifier, in the treatment of external genital/perianal warts in female patients. Imiquimod, which has antiviral properties, elevates the levels of cytokines such as interferon-alpha resulting in stimulation of cell-mediated immunity.
**Study design:** Females with external genital warts applied imiquimod 5% cream 3 times per week, for up to 16 weeks. Patients who cleared their warts entered a 6-month follow-up period. If their warts recurred, or new warts developed, patients could be re-treated for up to 16 additional weeks. Patients who experienced partial clearance in the initial treatment period could enter a second consecutive 16-week treatment period. Patients in this group who cleared their warts were also monitored for 6 months.
**Results:** A total of 410 female patients, from 114 clinic sites in 20 countries participated in this study. Complete clearance was observed in 59.3% of patients (intent-to-treat analysis) in the initial treatment period, with an additional 6.1% clearing in the second consecutive treatment period. The overall clearance rate was 65.4%. In a treatment failures analysis that excluded those patients who discontinued for reasons unrelated to safety or lack of efficacy, the clearance rate was 75.4%. Only 5.4% and 15.3% of patients experienced recurrence of warts in the 3- and 6-month follow-up periods, respectively, resulting in sustained clearance rates (patients who cleared during treatment and remained clear at the end of the follow-up period) of 55.6% and 45.8%. For patients who re-applied imiquimod for up to an additional 16 weeks after experiencing wart recurrence, the clearance rate was 67.7% (21/31).

Local skin reactions in the majority of patients were of mild to moderate severity; the most frequently reported was erythema, which occurred in 64% of patients. The frequency of local skin reactions decreased from initial to subsequent treatment periods.

**Conclusion:** Imiquimod 5% cream is a safe and effective treatment for external genital warts in females for up to 16 weeks and continues to provide a well-tolerated benefit to those treated for up to 32 weeks.

---

### Determination of *Ureaplasma urealyticum* biovars in post-partum endometritis (PPE)

*Chaim Walter, Horowitz Shulamith, Ingel Frida, Evinson Bela and Mazor Moshe*

*Department of Obstetrics and Gynecology and Department of Microbiology and Immunology, Soroka University Medical Center, Ben-Gurion University, Beer-Sheva, Israel*

**Objective:** There are two biovars (*parvo* and T960) comprising 14 serovars of *U. urealyticum* (1, 3, 6, 14 and 2, 5, 8, 9–13, respectively). Some serovars have been shown to be associated with disease syndromes (prematurity, recurrent abortions, urethritis, infertility etc.). Our objective was to determine whether there is a predominance of a certain biovar of *U. urealyticum* in postpartum endometritis (PPE).

**Study design:** *U. urealyticum* biovars as well as anti-ureaplasma antibodies were determined in a group of patients who had previously presented with PPE and who were culture positive for *U. urealyticum*.

**Results:** We have previously found that there is a significant difference between women with PPE and controls regarding level of colonization (cfu). In a larger study we found that 26 out of 67 (38.8%) of culture positive PPE patients had very high levels of *U. urealyticum* (> $10^5$ cfu/ml) compared to 5 out of 30 (16.7%) in the control group (puerperal women without PPE) ($p$ = 0.03). Determination of *U. urealyticum* biovars revealed that there was no significant difference between the PPE patients and the controls. Neither was there a statistically significant difference between the study and the control groups regarding anti-ureaplasma antibodies (30% vs 18%, respectively).

**Conclusions:** The involvement of *U. urealyticum* in the development of puerperal morbidity is dependent on the colonization rate in the cervix (> $10^5$ cfu/ml), and not merely on its presence or absence in a culture. The prevalence of the *U. urealyticum* biovars in the PPE group was similar to that in the control group.

---

### Antimicrobial resistance alert among anaerobes: comparison of obstetric-gynecological and intra-abdominal (IA) isolates

*K. E. Aldridge PhD and D. Ashcraft BS (MT)*

*LSU Health Sciences Center, New Orleans, LA*

**Objectives:** Recent studies have shown that patients who develop bacteremia with anaerobes (particularly of the *B. fragilis* group) (Bfg) and are receiving inappropriate empiric antimicrobial therapy have a significantly higher mortality rate[1,2]. In a recent *in vitro* study we compared the susceptibility rates of similar groups of anaerobes from obstetric-gynecological (Ob-Gyn) and intra-abdominal (IA) sources.

**Study design:** Using a broth microdilution method as recommended by the National Committee for Clinical Laboratories Standards (NCCLS), susceptibility MIC data were determined for seven antimicrobials. 112 Ob-Gyn and 346 IA isolates were tested; 46% and 81% were Bfg isolates, the remaining being isolates of *Prevotella* (Prev), *Fusobacterium* (Fuso), *Porphyromonas* (Por), and *Peptostreptococcus* (Pept).

**Results:** Against both Ob-Gyn and IA the most active agents were piperacillin/tazobactam (P/T), metronidazole (MET), and imipenem (IMP) (one Pept. isolate from each source was resistant to MET). Of the Ob-Gyn isolates 13% of the Bfg had reduced susceptibility to clindamycin (CL) compared to 30% for IA isolates. Resistance to CL was 13% for Pept and Prev isolates from Ob-Gyn but less (6% and 12%) for IA isolates. Ampicillin/sulbactam (A/S) resistance ranged from 0 to 33% for Bfg species and was 14% among Prev from Ob-Gyn isolates. For IA isolates A/S resistance ranged from 4 to 10% for Bfg and was 0% among Prev, Porph and Pept. Cefoxitin (FOX) resistance was 8% and 7% for Bfg isolates from Ob-Gyn and IA sources respectively, but no resistance was found among the other groups.

**Conclusions:** Antimicrobial resistance appears to be increasing among both Ob-Gyn and IA isolates to common antimicrobials; the Bfg plays a major role in this resistance and accounts for the majority of the isolates in each group. Increasing CL and A/S resistance may severely compromise their current and/or future use. These data underscore the changing resistance among clinically significant anaerobes and indicate which agents may be most useful empirically.

**References**

1. Redondo MC *et al. Clin Infect Dis* 1995;20:1492–6
2. Nguyen MH *et al. Clin Infect Dis* 2000;30:870–6

---

### Patterns of sexual activity among high-risk minority women: implications for topical microbicide development

*J. M. Piper MD, R. N. Shain PhD, A. E. Holden MA, S. Perdue DrPH and J. D. Champion PhD*

*Department of Obstetrics and Gynecology, UTHSC-SA, San Antonio, TX*

**Objectives:** Design of topical microbicides must consider the differing surfaces to which they may be applied. Products designed to prevent the transmission of sexually transmitted diseases (STDs) and human immunodeficiency virus (HIV) in women must be safe and effective in the vagina and possibly also the rectum and oral cavity. We sought to describe the sexual activity patterns of minority women with active STDs to determine the need for rectal and/or oral efficacy and safety of topical microbicides.

**Study design:** Minority women with an active STD (gonorrhea, chlamydia or *Trichomonas*) were questioned extensively about their recent (previous three months) and lifetime sexual activity. Sexual activity patterns (including use of protection) are described below, stratified by ethnicity (Hispanic *vs.* Black) and age (< 19 *vs.* ≥ 19).

**Results:** All 827 participants reported recent vaginal sex. Rates of reported anal and oral sex, both lifetime and recent, are described below, along with the percentage of women who recently engaged in each type and never used protection

**Table 1** Pattern of sexual activity in study group

| *Category* | *Overall* | *Hispanic* | *Black* | *Age < 19* | *Age ≥ 19* |
|---|---|---|---|---|---|
| Ever had anal sex | 23% | 24% | 20% | 15% | 30% |
| Ever had oral sex | 64% | 65% | 59% | 51% | 74% |
| Recent anal sex | 15% | 16% | 13% | 10% | 19% |
| Recent oral sex | 50% | 53% | 37% | 36% | 61% |
| No protection – vaginal sex | 40% | 42% | 33% | 38% | 41% |
| No protection – anal sex | 84% | 88% | 71% | 82% | 85% |
| No protection – oral sex | 94% | 94% | 92% | 95% | 93% |

(condoms or dental dams) with that type of sex (Table 1).

**Conclusions:** High-risk minority women commonly engage in non-vaginal sexual activity, with oral sex most common (~2/3) and anal sex reported by a substantial minority (~1/4). Use of protection for non-vaginal sex was rare. Although a topical microbicide safe and efficacious only for vaginal use would benefit women in our population, there is a great need for the development of microbicides that are also safe and effective for both oral and rectal use.

### Vaginal microflora of sexually inexperienced women: impact of sexual debut

*S. L. Hillier PhD, M. A. Krohn PhD, L. Meyn MS and D. V. Landers MD*

*University of Pittsburgh/Magee-Women's Hospital, Pittsburgh, PA*

**Objective:** To describe the vaginal microflora of sexually inexperienced women, and to assess the impact of sexual debut on the vaginal ecosystem.

**Study design:** Ninety-seven women aged from 18 to 30 who denied previous vaginal/penile intercourse were recruited from 3 clinical sites. Vaginal specimens were transported in Amies media for detection of group B *Streptococcus* (GBS), *Lactobacillus*, yeast and bacterial vaginosis (BV). Vaginal micro-organisms were detected by culture methods and BV was diagnosed using Gram-stained vaginal smears evaluated by the Nugent criteria. Follow-up cultures and data were available for 73 women at four-month intervals thereafter, for a total of 67.6 woman-years of follow-up.

**Results**: Nineteen (19.6%) of the virgins were colonized by GBS, 58 (59.8%) by hydrogen peroxide-producing lactobacilli, 20 (20.6%) by yeast, and 17 (17.5%) had Gram stain evidence of BV. Initiation of vaginal intercourse was reported by 27 (37.0%) of the 73 women followed for a year. The rates of acquisition of GBS, yeast and BV, and rate of loss of $H_2O_2$-producing lactobacilli stratified by sexual behavior are summarized in Table 1.

**Table 1** Rate of acquisition or loss of vaginal flora per 100 woman-years of follow-up (*$p < 0.05$)

| *Behavior* | *GBS* | *Yeast* | *BV* | *Loss of $H_2O$ + Lactobacillus* |
|---|---|---|---|---|
| *Recent sex* | | | | |
| Yes | 146* | 208* | 74* | 121 |
| No | 42 | 52 | 12 | 38 |
| *Unprotected sex* | | | | |
| Yes | 47 | 102 | 134* | 153* |
| No | 47 | 57 | 10 | 37 |

**Conclusions:** Initiation of vaginal intercourse leads to numerous changes in the vaginal microflora. Engaging in vaginal intercourse was associated with increased acquisition of GBS, yeast and BV, and unprotected intercourse was associated with increased acquisition of BV and loss of $H_2O_2$-producing lactobacilli.

### Demographic and behavioral risk factors for losing vaginal colonization with $H_2O_2$+ *Lactobacillus* spp.

*M. A. Krohn PhD, L. A. Meyn MS and S. L. Hillier PhD*

*Magee-Women's Hospital/University of Pittsburgh, Pittsburgh, PA*

**Objectives:** To determine demographic, behavorial, and sexual/hygiene factors that are associated with loss of vaginal colonization with $H_2O_2$+ *Lactobacillus* spp.

**Study design:** The study is a prospective, longitudinal, observational cohort study of 1248 non-pregnant women observed at 4 monthly intervals for 1 year from 1997 through 2001. Genital specimens and a behavorial questionnaire were done at each visit. Rates of lactobacilli loss were expressed per 100-woman years of follow-up and assessed for risk factors using Cox proportional hazard models. The values of exposure variables, assessed the visit before losing colonization, were used in the models.
**Results:** After adjusting for covariates, African-American women (HR = 1.5; 1.1–2.0); those with less than a high school education (HR = 2.5; 95% CI, 1.5–4.1); and women not using alcohol (HR = 1.5; 1.1–2.2) have elevated risks of losing *Lactobacillus* colonization. Women using no contraception (HR = 1.5; 1.02–2.1), women with intermediate flora (HR = 2.3; 1.7–3.1); those with BV (HR = 2.8; 2.0–3.9); and women with yeast (HR = 1.3; 1.03–1.8) had increased risks of losing *Lactobacillus*. Women using oral contraceptives had a 30% reduced risk of losing colonization compared with barrier users.
**Conclusions:** Surrogate demographic variables such as race and education affect *Lactobacillus* status by unknown mechanisms. However, proximal, modifiable factors such as contraceptive methods and other vaginal flora may influence the vaginal environment to the benefit or harm of *Lactobacillus* colonization.

---

### Interaction between lactobacilli and *Gardnerella vaginalis in vitro* as a key in the pathogenesis of bacterial vaginosis

*Alla Aroutcheva MD, PhD, Jose Simoes MD, PhD and Sebastian Faro MD, PhD*

**Objective:** To determine the factors produced by *Lactobacillus* that inhibit the growth of *Gardnerella vaginalis* and to study the ability of *G. vaginalis* to overcome lactobacilli.
**Study design:** Forty-one vaginal lactobacilli were studied for the production of acids, hydrogen peroxide, and bacteriocin. Forty-three *G. vaginalis* isolates were tested for growth in acid media, sensitivity to $H_2O_2$, bacteriocin, and inhibition effect on lactobacilli.
**Results:** Lactobacilli produce a large amount of organic acids that maintain a low pH in the vaginal environment. After 24 hours of growth the pH of *Lactobacillus* species in liquid MRS medium decreased from 6 to 5.1–4.0. Growth of *Lactobacillus* and *G. vaginalis* at different media pH was monitored with optical density (OD) within 48 h. Inhibition rate was calculated by subtracting the 48 h OD from the initial OD for each tested pH and control (pH 6.0). Taking the difference between control OD as 100% we calculated the percentage of inhibition. Media of pH 4.0 inhibited 82% of *G. vaginalis* growth and 37% of *Lactobacillus*; while at pH 4.5 the inhibition rates were 52% and 14% respectively. Eighty-two percent of the lactobacilli produced $H_2O_2$. Sensitivity of *G. vaginalis* to $H_2O_2$ ranged from < 15 to 100 µg/ml. The 72.7% of *Lactobacillus* isolates produced by bacteriocin inhibited growth of most of the *G. vaginalis* strains (77.8%). Sensitivity of *G. vaginalis* to purified bacteriocin (3.8 kDa) ranged between 12.5 and 0.75 µg/ml. Our study showed that *G. vaginalis* also produced factors bacteriostatic for *Lactobacillus*. Lysate from three *G. vaginalis* strains inhibited 79%, 73% and 55% of *Lactobacillus* growth in MRS broth at pH 6.0. At pH 4.0 the strains had decreased inhibition effects of 48.4%, 40.1% and 15%.
**Conclusion:** In the healthy vaginal ecosystem vaginal lactobacilli produce antibacterial factors that maintain *G. vaginalis* under suppressed conditions. However, *G. vaginalis* produces factors able to inhibit the growth of *Lactobacillus*. This mechanism probably takes place *in vivo* in cases of bacterial vaginosis.

---

### Do second-trimester amniotic fluid interleukin-6 and interleukin-10 concentrations predict preterm delivery?

*Joseph Apuzzio MD, Ying Chan MD, Nicholas Illsley PhD, Pei-Lin Kim MD and Stanley Vonhaggen PhD*

*New Jersey Medical School, Newark, NJ*

**Objective:** Interleukin-6 (IL-6) is an inflammatory cytokine that has been shown to be elevated in the amniotic fluid of patients with preterm labor, and interleukin-10 (IL-10) is an anti-inflammatory cytokine that has been shown to inhibit the synthesis of other cytokines. We hypothesize that amniotic fluid IL-10 in the early second trimester is low in patients who subsequently develop preterm labor. Because of its deficiency, excessive inflammatory responses associated with elevated levels of IL-6 lead to preterm labor and delivery.
**Study design:** Amniotic fluid IL-6 and IL-10 levels were measured in 96 women who had undergone genetic amniocentesis between 15 and 23 weeks' gestation. Levels of IL-6 and IL-10 were measured by immunoassay and correlated with demographic and pregnancy outcome information.
**Results:** Fifteen patients delivered at or before 36 weeks and 81 patients delivered after 36 weeks. There was an inverse correlation between amniotic fluid IL-6 concentration and gestational age at delivery. Similarly, an inverse correlation existed between amniotic fluid IL-10 concentration and gestational age at delivery.
**Conclusions:** Both IL-6 and IL-10 levels in the second-trimester amniotic fluid appear to be higher in patients who subsequently undergo preterm delivery. Therefore, low amniotic fluid IL-10 production during the second trimester does not seem to be an etiology for preterm labor.

---

### Tumor necrosis factor-alpha (TNF-a) down-regulates oxytocin receptor binding in cultured human uterine myocytes

*P. N. Rauk MD*

*Magee-Women's Research Institute and Department of Obstetrics, Gynecology, and Reproductive Sciences, University of Pittsburgh School of Medicine, Pittsburgh, PA*

**Objective:** Oxytocin receptor (OTR) increases in the myometrium immediately prior to the onset of both term and preterm labor. Intrauterine infection accounts for as much as 70% of all cases of preterm labor. Intrauterine infection results in the production of decidual inflammatory cytokines, specifically TNF-$\alpha$ and interleukin-1 and -6, which stimulate prostaglandin production and labor. We have previously demonstrated that IL-1 down regulates the OTR in human myometrium, while IL-6 up-regulates the OTR[1,2]. The objective of this study was to evaluate the effect of TNF-$\alpha$ on OTR in the human uterus.
**Study design:** Human uterine smooth muscle from term unlabored uteri were established in primary tissue culture. Cells were treated with TNF-$\alpha$ at increasing concentrations (0–20 ng/ml) and for various times (0–24 hours). Oxytocin receptor binding was measured using $^{125}$I-labeled oxytocin antagonist ornithine vasotocin (OVT). Oxytocin receptor mRNA was measured using semi-quantitative reverse transcription polymerase chain reaction (RT-PCR). Comparison between TNF-$\alpha$ treated cells and controls was made using ANOVA.
**Results:** The addition of TNF-$\alpha$ (0.1 and 1 ng/ml) resulted in a 25% and 50% reduction in OVT binding at 24 hours respectively. Receptor concentration was $200 \pm 80$ fmol/mg protein with TNF-$\alpha$ (1 ng/ml) compared to $558 \pm 200$ in treated control cells ($p < 0.05$). In time-course experiments loss of binding was exponential after 8 hours of treatment. RT-PCR experiments demonstrate a 50% reduction in oxytocin receptor mRNA beginning at 6 hours of TNF-$\alpha$ treatment.

**Conclusions:** These data demonstrate a sensitive time- and dose-dependent down-regulation of oxytocin receptor binding and oxytocin receptor mRNA by TNF-$\alpha$ in the human uterus. The down-regulation of prematurely induced uterine oxytocin receptors by TNF-$\alpha$ may be a protective mechanism limiting the preterm labor process in early infection. With prolonged infection other cytokines, especially IL-6, may subsequently induce the expression of OTR.

### References

1. Rauk PN, Friebe-Hoffman U. Interleukin-1$\beta$ down-regulates the oxytocin receptor in cultured uterine smooth muscle cells. *Am J Reprod Immun* 2000;43:85–91
2. Rauk PN, Friebe-Hoffman U, Winebrenner L, Chiao JP. Interleukin-6 up-regulates the oxytocin receptor in cultured uterine smooth muscle cells. *Am J Reprod Immun* 2001;45: 148–53

### Tumor necrosis factor-alpha (TNF-a) concentration in amniotic fluid and fetal death in a rabbit model of chronic infection

*M. Kunze[1], J. K. Davies[1], R. S. McDuffie[2], D. M. Wolf[1], M. P. Sherman[3], K. K. Leslie[1] and R. S. Gibbs[1]*

*[1]Department of Obstetrics and Gynecology, University of Colorado School of Medicine; [2]Kaiser Permanente, Denver, CO; and [3]Department of Pediatrics, University of California, Davis School of Medicine*

**Objectives:** Tumor necrosis factor-alpha (TNF-α) has been implicated as a mediator of septic shock and death, and has been detected in human amniotic fluid during preterm labor due to intra-amniotic infection. We investigated the correlates of fetal death in a pregnant rabbit model using inoculation of *Escherichia coli* with delayed antibiotic therapy.

**Study design:** As reported previously (Gibbs *et al.*, *IDSOG*, 2000), New Zealand white rabbits at 70% gestation were inoculated intracervically with $10^3$–$10^4$ cfu *E. coli* per uterine horn. We also inoculated two controls with saline. At varying intervals after inoculation (0.5–4.0 h), antibiotic therapy was initiated with ampicillin-sulbactam. Necropsy was performed after 72–120 hours. Uterus, placenta, decidua and fetal tissues were collected for culture. Histology was performed using a semiquantitative histologic inflammation (HI) score (based upon inflammation, congestion and edema). A measure of fetal death was expressed as the ratio of number of dead pups *in utero* divided by the total number of pups *in utero* (data not reported in *IDSOG* 2000). Amniotic fluids from these animals were analyzed for TNF-α levels by bioassay (data not reported). Logistic regression was carried out using SAS PROC GENMOD.

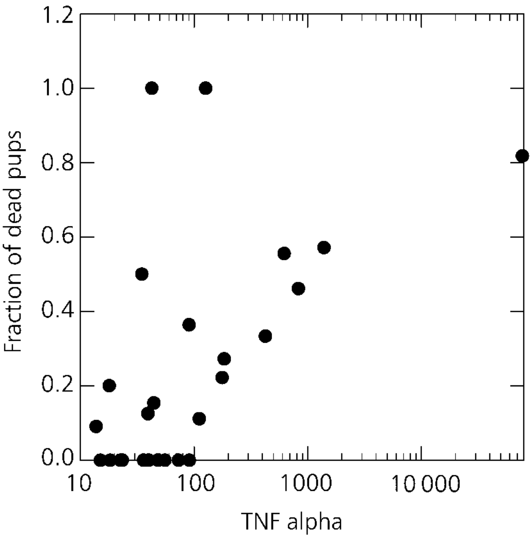

**Figure 1** Fraction of dead pups against TNF-α level

**Results:** Mean TNF-α levels in amniotic fluid were 10–1000-fold greater than those in the saline controls. The ratio of dead pups to total pups was correlated with log TNF-α values in amniotic fluid (see Figure 1). The only significant predictors of fetal death were log TNF-α ($p < 0.0001$) and uterine histologic inflammation score ($p < 0.0004$), but not positive culture of amniotic fluid, uterus, placenta or the fetus.

**Conclusions:** In a model of chronic infection induced by delayed antibiotic therapy, fetal death is independently correlated with log TNF-α level and uterine HI score, but not with culture status. Fetal death in this setting is not associated with infection *per se*, but rather with the host's response to infection.

---

### Decreased cervical proinflammatory cytokines in early pregnancy are associated with subsequent intra-amniotic infection

*H. N. Simhan MD, M. A. Krohn PhD, S. N. Caritis MD, B. Martinez de Tejada MD, D. V. Landers MD and S. L. Hillier PhD*

*University of Pittsburgh/Magee-Women's Hospital, Pittsburgh, PA*

**Objective:** To determine the association between the concentration of cervical proinflammatory cytokines early in pregnancy and the subsequent development of clinical intra-amniotic infection (IAI) in labor.

**Study design:** We enrolled 517 women from February 1996 to February 1997, of whom 403 had complete data. Two cervical swabs were collected at 8–20 weeks' gestation for the assay of interleukin-1β (IL-1β), interleukin-6 (IL-6), and interleukin-8 (IL-8). Cytokine concentration was measured by a commercially available enzyme linked immunosorbent assay (ELISA). Swabs were tested for *Trichomonas vaginalis*, *Chlamydia trachomatis, Neisseria gonorrhoeae*, bacterial vaginosis, and Group B *Streptococcus*. IAI was diagnosed by a temperature higher than 38° C and two of the following signs: maternal/fetal tachycardia, uterine tenderness, foul smelling vaginal discharge, and white blood cell count (WBC) > 18 000. The univariate and adjusted odds ratios for the development of IAI by cytokine concentrations lower than the first quartile were determined using logistic regression analysis. Several demographic, clinical, and infectious factors were assessed for effect-modification and confounding.

**Results:** Median concentrations of IL-1β, IL-6 and IL-8 are significantly decreased among women who subsequently develop IAI (Table 1 and Figure 1).

**Conclusions:** Decreased concentrations of cervical proinflammatory cytokines early in pregnancy are strongly associated with the development of subsequent IAI in labor, even when adjusted for obstetric and infectious risk factors.

**Table 1** Statistical analysis of the data

| *Cytokine* | *Unadjusted OR* | *Adjusted OR** | *95% CI* | *p* |
|---|---|---|---|---|
| IL-1β < first quartile | 3.00 | 3.41 | 1.47–7.89 | 0.004 |
| IL-6 < first quartile | 2.66 | 2.52 | 1.14–5.55 | 0.022 |
| IL-8 < first quartile | 2.34 | 2.60 | 1.20–5.66 | 0.016 |

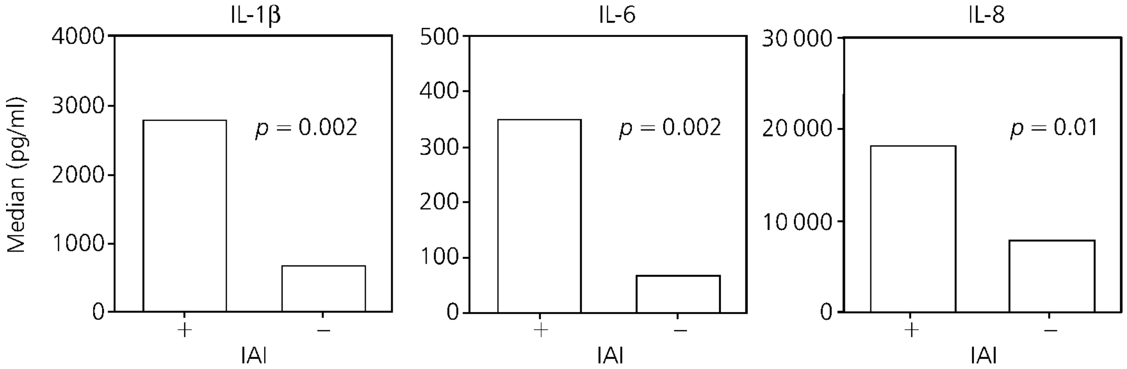

**Figure 1** Median concentrations of interleukins IL-1β, IL-6 and IL-8; IAI, intra-amniotic infection

### Relationship between maternal and fetal antibodies to vaginal anaerobes and preterm birth

*K. A. Boggess, S. Lieff, A. P. Murtha, P. Madrianos, J. Beck and S Offenbacher*

*University of North Carolina and Duke University, Chapel Hill, NC*

**Objective:** Our objective was to determine whether maternal or fetal humoral responses to vaginal anaerobes are associated with preterm birth (PTB).

**Study design:** A prospective cohort of pregnant women was enrolled at < 26 weeks' gestation. Maternal demographic and medical information was chart abstracted. Maternal serum was collected at enrollment and at delivery and analyzed for IgG to *Gardnerella vaginalis*, *Prevotella bivius*, *Mobiluncus curtsii*, and *Bacteroides urealyticus*. Umbilical cord serum was collected following delivery and analyzed for IgM to the same organisms. PTB was defined as delivery at less than 37 weeks as a result of preterm labor or premature rupture of membranes. Correlation was performed using Spearman rank correlation.

**Results:** 293 (78.1%) of 375 maternal samples collected at enrollment were IgG+ for any organism, compared to 194 (49.1%) of395 samples collected at delivery. Demographic characteristics were similar between antibody-positive and antibody-negative women with regard to age,

**Table 1** Rates of preterm birth (PTB) correlated with antibody status

| *Rate of PTB* | | *Rate of PTB* | | *OR (95% CI)* |
|---|---|---|---|---|
| Maternal antibody+ at enrollment | 78% | Maternal antibody– at enrollment | 22% | 1.0 (0.59–1.8) |
| Maternal antibody+ at delivery | 38% | Maternal antibody– at delivery | 62% | 0.64 (0.45–0.92) |
| Fetal antibody+ | 44% | Fetal antibody– | 56% | 0.66 (0.45–0.97) |

insurance marital status, parity and tobacco use. The presence of maternal antibody at enrollment was correlated with maternal antibody at delivery ($r = 0.59$, $p = 0.04$). There was a negative correlation between antibody at delivery and BV during pregnancy ($r = -0.36$, $p = 0.04$). The presence of maternal antibody at enrollment or delivery was negatively associated with fetal antibody ($r = -0.34$, $p = 0.06$ and $r = -0.41$, $p = 0.05$, respectively). The rate of PTB of the entire cohort was 26%. Women who were antibody+ at delivery or whose fetuses were antibody+ were less likely to have PTB (Table 1).

The PTB rate was highest in women who were antibody-positive at enrollment and whose fetuses were antibody-negative (49%).

**Conclusion:** Exposure to vaginal anaerobes with an inadequate maternal or fetal humoral response may be related to the causal mechanism between BV and prematurity.

**Acknowledgement**

This research was supported by NIDCR grant RO-1-DE-12453, P-60-DE-13079 and T32-DE-07310.

---

### Does the clinical setting influence vaccine awareness in obstetric and gynecology patients?

*Bernard Gonik MD and Mark Tomlinson MD*

*Department of Obstetrics and Gynecology, Wayne State University School of Medicine, Detroit, MI and Women's Healthcare Association, Portland, OR*

**Objective:** To better understand patient vaccine awareness in different clinical settings.

**Study design:** A survey was completed by patients at an obstetric and gynecology resident teaching clinic (CLINIC) ($n = 228$) and by private community physician patients (PVT) ($n = 254$). The masked questionnaires contained yes/no or multiple choice questions exploring demographic, immunization recollection and vaccine administration preferences for these two study populations. Chi-square for categorical variables and Student's *t*-test for continuous variables were used for statistical analyses.

**Results:** The CLINIC group were younger than the PVT group (27.2 ± 10.6 yrs *vs*. 38.0 ± 12.6 yrs; $p < 0.001$) and presented more often for pregnancy-related visits (57.0% *vs*. 21.6%; $p < 0.001$). PVT more commonly had documentation of childhood (34.3%) and adult (26.8%) vaccine status than CLINIC (25.6% and 15.5%, respectively) ($p < 0.03$). Of the vaccine-preventable diseases (VPD) surveyed, PVT (*vs*. CLINIC) more often reported adequate vaccination or prior exposure to rubella (68.5% *vs*. 30.5%; $p < 0.001$) and varicella (65.7% *vs*. 48.5%; $p < 0.001$). No differences were noted between the study groups for hepatitis B, tetanus, and influenza. The adequacy of vaccination for these latter VPD was below 50% for both PVT and CLINIC groups. The PVT group more often identified a non-obstetric/gynecological primary care provider for vaccine-related needs, as opposed to the CLINIC group, who more often relied on their obstetric and gynecological or health department sites. Over a third of both populations could not identify a provider for vaccine administration. Both The PVT group (78.8%) and the CLINIC group (84.0%) strongly desired availability of vaccine services through their obstetric and gynecological office.

**Conclusions:** Private community physician patients demonstrate a better awareness of vaccine status than do those attending a teaching clinic. However, overall both populations report poor documentation and inadequate immunity against most VPD. A variety of practice sites are currently utilized for vaccination, although many patients cannot identify a place to go. The majority of patients would like to see this service available as a part of their obstetric and gynecological care.

### Point mutation in *Escherichia coli* Dr fimbriae abolishes type IV collagen-binding and renal persistence in experimental chronic pyelonephritis

*Rangaraj Selvarangan[1], Pawel Goluszko[1], Jyotsana Singhal[1], Christophe Carnoy[2], Steve Moseley[3], Billy Hudson[4], Stella Nowicki[1] and Bogdan J. Nowicki[1]*

*[1]Departments of Obstetrics and Gynecology and Microbiology and Immunology, UT Med. Branch, Galveston, TX-77555-1062; [2]Institute de Biologie de Lille, Lille, FRANCE; [3]Univesity of Washington, Seattle, WA; [4]University of Kansas Medical Center, Kansas city, KS*

**Introduction:** The role of *Escherichia coli* virulence factors in recurrent and chronic infections of the urinary tract is poorly understood. The Dr fimbriae of *E. coli* display unique tropism to the renal interstitium enabling the bacterium to cause experimental chronic pyelonephritis in mice. The renal receptors for $Dr^+$ *E. coli* are decay-accelerating factor (DAF) and type IV collagen. While the total loss of Dr fimbrial binding activity resulted in prevention of experimental chronic pyelonephritis, the contribution of the individual receptors to the chronic infectious process requires further investigation. The apical cellular receptor, DAF mediates colonization and internalization of the $Dr^+$ *E. coli* into epithelial cells.

**Objectives:** We hypothesized that the access of bacteria to the basement membrane collagen provides protected colonization sites for $Dr^+$ *E. coli* and promotes chronicity.

**Study design:** To test this hypothesis and the role of type IV collagen in the renal persistence of $Dr^+$ *E. coli* we constructed an isogenic mutant I113T in the DraE adhesin subunit retaining DAF binding but unable to bind type IV collagen. The mutant lost interstitial binding to both human and mouse kidneys. The parent strain *E. coli* IH11128 and its collagen non-binding isogenic mutant I113T were evaluated for persistence of infection in an established model of tubulo-interstitial nephritis in C3H/HeJ mice. Groups of mice were infected intravesically and sacrificed at different time points for quantitative culture of the kidneys.

**Results:** The mice infected with collagen mutant Dr-I113T eliminated infection by 8 weeks while the parent strain caused persistent infection that lasted at least 14 weeks ($p \leq 0.02$). A trans-complementation experiment that restored type IV collagen and kidney-binding also restored the capacity to cause persistent infection. We conclude that DraE-mediated type IV collagen binding is necessary for the development of chronic pyelonephritis in a murine model of *E. coli* urinary tract infection.

### Perioperative antimicrobial prophylaxis can be improved with regard to urinary tract infection by means of a long-acting cephalosporin such as ceftriaxon

*UB Hoyme and Katrin Schönheit*

*Department of Gynaecology and Obstetrics, Klinikum Erfurt, Germany*

**Objectives:** Having determined the requirement for antimicrobial prophylaxis in both obstetrical and gynecological procedures most studies demonstrated similar efficacy irrespective of agent or number of doses. For that reason we sought to ascertain any difference in urinary tract infection associated with the half life of pharmakon.

**Study design:** In a prospective randomized comparative study 2 g mezlocillin (Baypen, Bayer, Leverkusen) ($n = 250$), 2 g cefotiam (Spizef, Takeda/Grünenthal, Aachen) ($n = 299$) or 1 g ceftriaxon (Rocephin, Roche, Grenzach-Wyhlen) ($n = 263$) was administered iv as perioperative prophylaxis for women undergoing obstetrical or gynecological surgical procedures.
Results: There was a 6.0% overall prevalence of wound infection with no difference between the study groups. A total of 136 women acquired a urinary tract infection: 21.6% following mezlocillin vs. 16.4% following cefotiam and 12.5% in the ceftriaxon group ($p < 0.05$).
Postoperative antibiotics were administered in 21.9% of all patients (88.0% because of urinary tract infection): 25.6% in the mezlocillin group vs. 24.1% following cefotiam, and 16.0% after treatment with ceftriaxon ($p < 0.5$).
**Conclusion:** The rate of postoperative urinary tract infections could be reduced following prophylaxis with the long-acting third generation cephalosporin ceftriaxon in comparison to standard antibiotics. This led to a further reduction in the use of antibiotics for postoperative complications.

---

### Progression of periodontal disease during pregnancy is associated with an increased risk for preterm delivery

*A. P. Murtha[1], S. Lieff[2], D. E. Abel[1], R. P. Heine[1], K. A. Boggess[2], H. L. Jared[2] and S. Offenbacher[2]*

*[1]Department of Obstetrics and Gynecology, Duke University Medical Center, Durham, NC; [2]UNC School of Dentistry, Chapel Hill, NC*

**Objective:** Preliminary data suggest an association between periodontal disease and preterm birth. Our objective was to determine whether the progression of periodontal disease during pregnancy is associated with an increase risk for preterm birth.
**Study design:** The Oral Conditions and Pregnancy study is a prospective study to examine the relationship of preterm birth to periodontal disease. Oral examinations are performed prior to 26 weeks' gestation and repeated within 48 hours of delivery. Pocket depth was measured at 6 sites on each tooth with a periodontal probe. Periodontal disease was defined as 4 sites with ≥ 5 mm pocket depth. Progression of periodontal disease was defined as 4 sites with ≥ 2 mm increase in pocket depth. Subjects with periodontal disease at baseline were divided into those with and without progression. Preterm birth was defined as delivery prior to 37 weeks. Data were analyzed using Chi square and logistic regression.
**Results:** Of the 722 subjects who completed both oral exams, 88 had periodontal disease at the baseline exam. Of these 88 subjects 32 (36.4%) had progression of periodontal disease during pregnancy. The preterm birth rate was significantly higher in the presence of progression when compared to those without progression (47% *vs.* 27%, $p = 0.04$). After controlling for potential confounders including race, marital status and prior preterm birth an odds ratio of 4.97 (1.5–16.1) was obtained. The adjusted odds ratio for delivery prior to 35 weeks in women with periodontal disease progression was 5.94 (1.5–22.9).
**Conclusions:** Progression of periodontal disease during pregnancy is associated with a five-fold rise in the risk for preterm birth. Progression of periodontal disease is associated with an increased local inflammatory response and infectious burden that may be manifest not only as a local progression of the disease but also as systemic effects ultimately leading to preterm birth. Further research to establish a causal pathway is in progress.

### Acknowledgement

This research was supported by NIDCR grants RO-1-DE-12453, P-60-DE-13079 and T32-DE-07310.

### Histologic examination of the umbilical cord as an indicator of intrauterine inflammation and neonatal morbidity among preterm infants

*Carolyn Gardella MD, Jane Hitti MD, MPH, Dorothy Patton PhD and David Eschenbach MD*

*Department of Obstetrics and Gynecology, University of Washington, Seattle, WA*

**Objectives:** To describe the histopathology of the umbilical cord as it relates to intrauterine infection and inflammation and to determine if funisitis is associated with neonatal morbidity among preterm infants.
**Study design:** A cohort of 115 afebrile women with preterm labor and intact membranes who ultimately delivered at less than 34 weeks had amniotic fluid (AF) collected by transabdominal amniocentesis for culture and for interleukin (IL)-6, IL-8 and tumor necrosis factor-alpha (TNF-α) levels. After delivery the chorioamnion and umbilical cord histology were examined and neonatal outcomes were recorded. Funisitis was systematically graded based on the presence of ≥ 5 neutrophils in the umbilical vessels or Wharton's jelly (0 = none, 1 = present in 1 vessel, 2 = present in 2–3 vessels, 3 = present in Wharton's jelly). AF and chorioamnion characteristics and neonatal outcomes were compared by the grade of funisitis.
**Results:** The results are shown in Table 1.
**Conclusions:** Funisitis grade > 0 was strongly associated with indicators of AF inflammation and histologic chorioamnionitis. Neonatal morbidity was associated with grade of funisitis. The risk of neonatal morbidity was greatest among infants with the higher grades of funisitis relative to those without funisitis, but these associations did not reach statistical significance. Histologic evaluation of the umbilical cord is technically easy and may have clinical applications to provide information about the intrauterine milieu retrospectively. Further study of the association between funisitis and neonatal morbidity is warranted.

### Acknowledgement
This research was supported by NIH grant R01 AI-31871.

**Table 1** Histology of umbilical cord and chorioamnion correlated with presence of neutrophils

| | *Grade of funisitis* | | | | |
|---|---|---|---|---|---|
| | *0* (n = *84*) | *1* (n = *7*) | *2* (n = *13*) | *3* (n = *11*) | *p** |
| *Amniotic fluid (%)* | | | | | |
| Culture+ | 6 | 57 | 23 | 46 | < 0.001 |
| IL-6 > 2000 pg/ml | 19 | 71 | 54 | 80 | < 0.001 |
| TNF-α > 30 pg/ml | 10 | 71 | 46 | 50 | < 0.001 |
| Culture+ or cytokine+ | 33 | 71 | 62 | 90 | 0.001 |
| *Chorioamnion (%)* | | | | | |
| Culture+ | 8 | 29 | 8 | 18 | 0.3 |
| Histologic chorioamnionitis | 21 | 100 | 85 | 82 | < 0.001 |
| Necrotic membrane roll | 14 | 17 | 23 | 46 | 0.2 |
| *Neonatal outcome* | | | | | |
| Delivery EGA (median, wks) | 31 | 31 | 28 | 29 | 0.1 |
| Birth weight (median, g) | 1700 | 1542 | 1351 | 1439 | 0.1 |
| Any morbidity†, % | 50 | 43 | 75 | 90 | 0.04 |
| RR (95% CI) | (Ref) | 0.4 (0.1–1.3) | 1.4 (0.4–4.6) | 4.2 (0.8–21.5) | |
| aRR (95% CI) | (Ref) | 0.3 (0.1–1.4) | 1.3 (0.3–5.3) | 3.6 (0.6–21.2) | |

*$\chi^2$ or Kruskall Wallis, as appropriate; †includes: RDS, sepsis, NEC, grade 3 or 4 IVH, or death; RR, relative risk; aRR, relative risk adjusted for birth weight

### Cerebral palsy, *Escherichia coli* and group B *Streptococcus*

*H. M. McDonald[1] PhD, S. Aitchison[2] BSc, J. E. Hiller[2] PhD, T. Y. Khong[1] FRCPA, J. S. Robinson[2] FRACOG and R. Vigneswaran[1]FRCP*

*[1]Women's & Children's Hospital (WCH), Adelaide, Australia; [2]University of Adelaide, Adelaide, Australia*

**Objective:** To determine the association between chorioamnionitis and cerebral palsy (CP) in very low birthweight infants.
**Study design:** In a case-cohort study all very low birthweight (VLBW) infants born at the WCH who developed CP that was confirmed at 5 years of age, were included ($n = 82$). The comparison group (sub-cohort) was a random sample of infants from the WCH VLBW database, which follows infants from birth to seven years of age. As this random sampling did not exclude CP cases, 24 infants who had CP were also randomly chosen for the sub-cohort and therefore formed a third group in the analysis. Selection criteria for the database changed slightly over the time period of the study, and selection of the sub-cohort was stratified to reflect these changes. More than 92 variables were analyzed and related to the occurrence of CP. Histopathological examination ($n = 175$) and microbiological culture ($n = 90$) of the placenta (interface between amniochorionic membranes) were performed.
**Results:** Threatened preterm labor without preterm delivery (TPTL), neonatal sepsis, placental *E. coli* and group B Streptococcus (GBS) and were significantly associated with CP (Table 1).
**Conclusion:** Very low birthweight infants with *E. coli* or group B *Streptococcus* infection of the amniochorionic membranes have more than four times the risk of CP.

**Table 1** Histological and microbiological findings

| | *CP not sub-cohort, n = 58* | *Subcohort not CP, n = 183* | *CP & sub-cohort, n = 24* | *Hazard ratio* | *95% CI* |
|---|---|---|---|---|---|
| Clinical chorioamnionitis | 6/58 | 24/183 | 3/24 | ns | |
| Histological chorioamnionitis | 10/33 | 37/124 | 10/18 | ns | |
| TPTL | 11/58 | 14/183 | 4/24 | 2.2 | 1.1–4.5 |
| Placental *E. coli* | 6/19 | 3/61 | 0/10 | 4.6 | 1.2–18.5 |
| Placental GBS | 3/19 | 3/61 | 2/10 | 4.1 | 1.3–12.9 |
| Neonatal sepsis | 13/58 | 25/183 | 5/24 | 2 | 1.0–4.0 |

### 'Real world' compliance with strategies to prevent early onset group B streptococcal disease

*L. Riley MD, M. Apollon MD, S. Haider MD, MPH, S. Chan-Flynn CNM, M. Rein MD, A. Cohen, J. Ecker MD and E. Lieberman MD, DrPH*

*Harvard Medical School*

**Objective:** To assess the 'real-world' compliance with risk-based and culture-based strategies to prevent early onset group B Streptococcal (GBS) disease.
**Study design:** We reviewed the medical records of consecutive term pregnancies delivered at 3 institutions (hospitals A and B were academic institutions and hospital C a community hospital) between January and March 1998. We abstracted demographic data, risk factors for GBS, GBS culture information, intrapartum antibiotic prophylaxis (IAP) and adverse drug reactions.
**Results:** At hospital A ($n = 314$), where the culture-based strategy is employed, 297 women (94.6%) had GBS cultures performed between 35 and 37 weeks' gestation and documented in the labor record. Of 58 women with positive GBS cultures, all received IAP. Of 17 women of unknown

GBS status, 3 had risk factors and received IAP. At hospital B ($n$ = 335), where the risk-based strategy is employed, 53 women (15.8%) had risk factors for GBS, of whom 48 (90.6%) were treated appropriately with IAP. At hospital C ($n$ = 178), 116 women had risk-based strategies applied and 62 had the culture-based strategy. Of women in the risk-based strategy, 20 (17.2%) had risk factors and 19/20 (95%) received IAP. Of women in the culture-based strategy, 38 (61.3%) had culture status documented in labor. Nine women with positive cultures received IAP. Of 27 with negative cultures, 5 (18.5%) received IAP. Among 24%, 18.8% and 19.1% of women receiving antibiotics at hospitals A, B and C respectively, only one minor adverse drug reaction (hives) was noted.

**Conclusions:** Compliance with the risk-based strategy was 91.8 % while compliance with the culture-based strategy was 100 %. There was a tendency to combine strategies in the culture-based approach leading to an overuse of antibiotics.
